# Impact of Primary Tumor Resection on Type B Lactic Acidosis in a Case of Metastatic Colon Cancer

**DOI:** 10.7759/cureus.62932

**Published:** 2024-06-22

**Authors:** Ankur Cheleng, Mithilesh K Sinha, Soumyajit Jana

**Affiliations:** 1 General Surgery, All India Institute of Medical Sciences, Bhubaneswar, Bhubaneswar, IND

**Keywords:** warburg effect, metastasis, hyperlactatemia, lactic acidosis, colon cancer

## Abstract

Malignancies seldom lead to hyperlactatemia or lactic acidosis. The elimination of the primary tumor is anticipated to result in the amelioration of lactate levels in such situations. A patient with obstructing descending colon cancer was subjected to surgical intervention as their serum lactate levels reached 3.6 mmol/L. The tumor was removed, and the ischemic bowel proximal to it was excised as well. The patient demonstrated signs of recuperation; however, their serum lactate levels persisted at levels exceeding 6.5 mmol/L. Consequently, the patient was subjected to further investigation and surgical intervention. A CT scan of the brain and abdomen indicated metastases to the liver and brain, respectively. The presence of metastases in colonic malignancies may impede the normalization of hyperlactatemia even after excising the primary tumor. The interpretation of lactate levels can be challenging and radiological assessments, including abdominal reexploration, may be required to ascertain the diagnosis.

## Introduction

Type B lactic acidosis is a rare complication of solid malignancies, with an unclear understanding of its mechanism [1,2]. While chemotherapy has been shown to decrease serum lactate levels, the effect of surgical intervention remains uncertain [2,3]. In this case report, we aim to analyze the outcome of the excision of the primary tumor on raised lactate levels in a patient with type B lactic acidosis.

## Case presentation

A 52-year-old female patient presented to the emergency department with the chief complaint of abdominal pain for one week. The pain was constant, diffuse throughout the abdomen, and not alleviated by medication. As time progressed, the patient's abdomen became distended, and she experienced several episodes of vomiting that contained undigested food. The patient had a history of hypertension and hypothyroidism, but she was currently taking prescribed medications for these conditions. During the examination, the patient was conscious and fully oriented to time, place, and person, with a Glasgow Coma Scale (GCS) score of 15/15. Her pulse rate was 117 beats per minute and her blood pressure was 90/60 mmHg. The abdomen was distended, and there was the presence of diffuse guarding along with tenderness. The bowel sounds were not present. A per-rectal examination did not reveal any palpable growths or masses.

The USG of the entire abdomen indicated an abrupt, short segment narrowing with thickening of the circumferential wall in the descending and sigmoid colon, along with distension of the proximal bowel. An erect abdominal X-ray also revealed dilated large bowel loops. Routine blood tests showed a hemoglobin level of 10.6 gm/dL and a white blood cell count of 15.4 x 10^3^/cumm (Table 1). An arterial blood gas analysis revealed a blood pH of 7.4 but a serum lactate level of 3.6 mmol/L (Table 2).

**Table 1:** Lab test results on admission *as per the reference range values used in the institution (All India Institute of Medical Sciences, Bhubaneswar)

Investigations	Result	Unit	Reference Range*
Hemoglobin	10.6	gm/dL	13-17
White Blood Cell Count	15.4	*10^3^/cumm	4-11

**Table 2:** Arterial blood gas analysis results on admission *as per the reference range values used in the institution (AIIMS, Bhubaneswar)

Test	Value	Unit	Reference Range*
pH	7.4	-	7.35-7.45
Lactate	3.6	mmol/L	< 2

**Figure 1:**
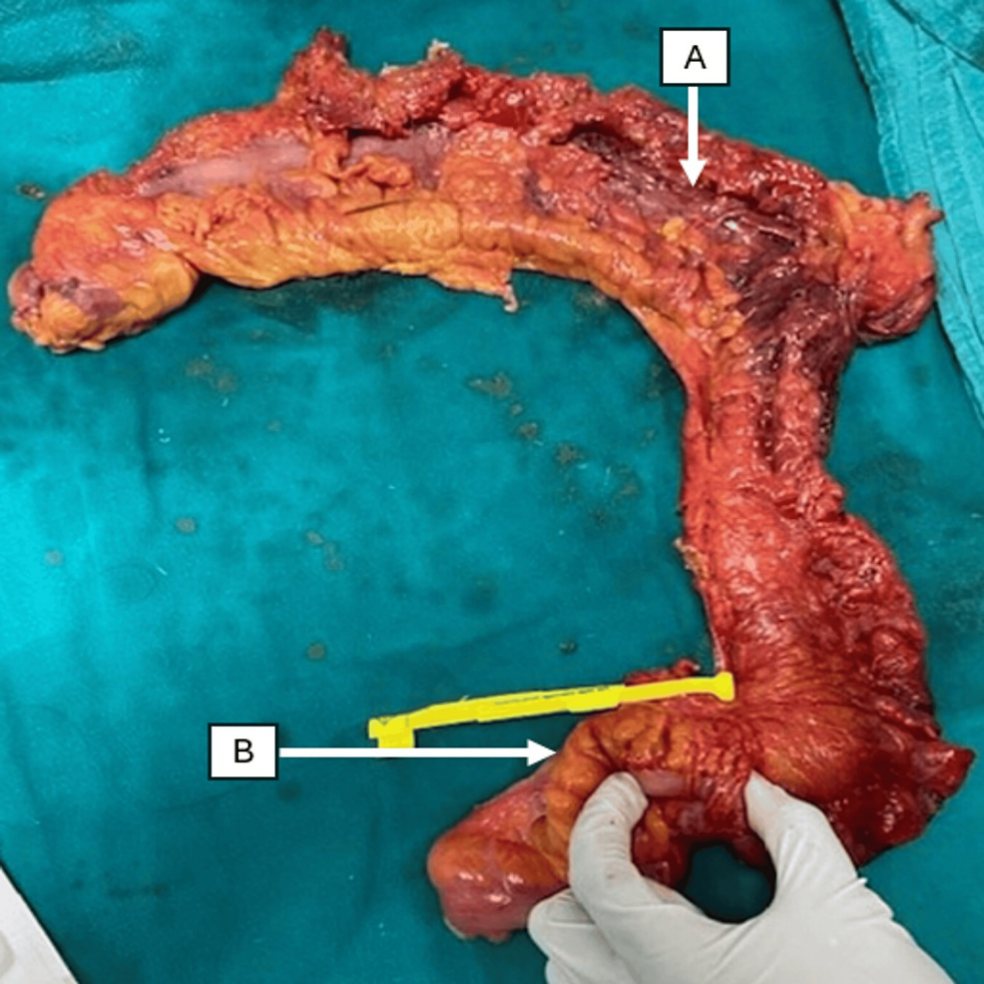
Resected specimen following left hemicolectomy showing gangrenous transverse colon with few perforations (A) and a rectosigmoid growth (B)

A provisional diagnosis of large bowel obstruction because of growth in the rectosigmoid area was made, and the decision to proceed with an exploratory laparotomy was taken as clinically she was in sepsis and had features of peritonitis. Multiple gangrenous patches and perforations were observed during the surgery in the transverse and descending colon. A solid mass measuring 4x4 cm was discovered at the junction of the rectum and sigmoid colon, and ascites were present, along with multiple secondary tumors in the liver (Figure 1).

A left hemicolectomy with end transverse colostomy was performed.

**Figure 2:**
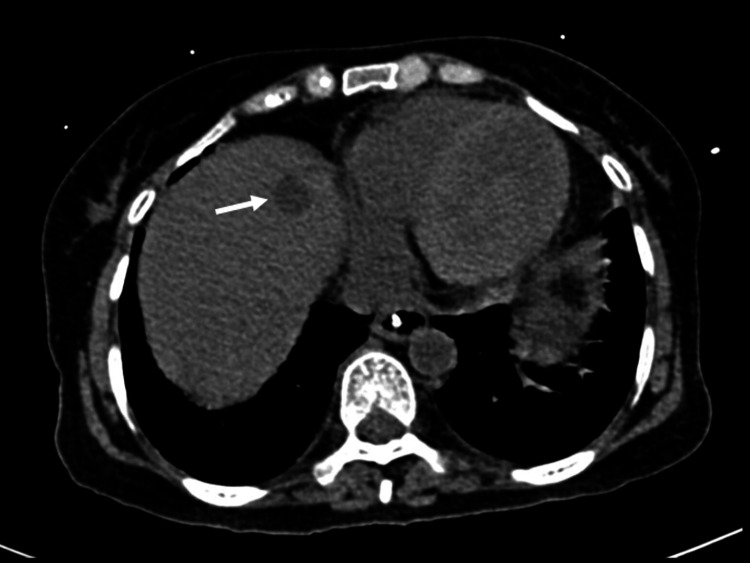
Contrast-enhanced CT (CECT) scan of the abdomen showing liver metastasis

**Figure 3:**
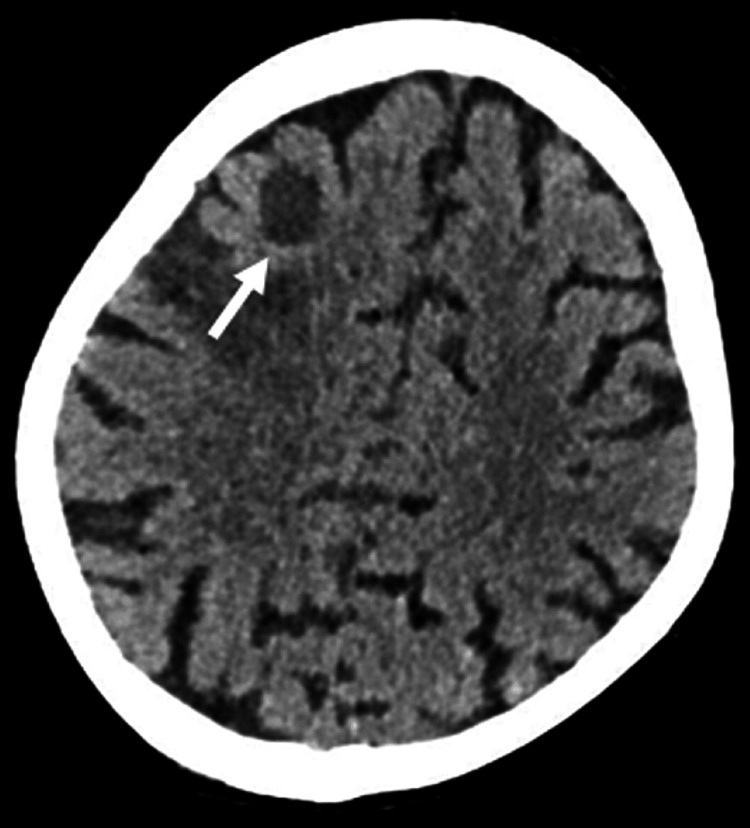
Non-contrast-enhanced CT (CECT) scan of the brain showing brain metastasis

Following the surgery, the patient's vital signs were stable, and the stoma began functioning. Intensivists made attempts to extubate the patient, but they were unsuccessful as there was no spontaneous breathing effort. Meanwhile, arterial blood gas analysis consistently showed high values of more than 6.5 mmol/L for serum lactate, accompanied by a drop in pH value to less than 7.35 on some occasions. The abdominal pressure was measured and found to be 12 cm of water. The total leukocyte count of 11.7x109 /L was within the normal range, but the differential count of neutrophils had increased to 95%. To eliminate the possibility of an intra-abdominal collection, an ultrasound of the abdomen was performed, which revealed mild intrabdominal fluid. On the fourth day after surgery, because of the persistently increased lactate levels, a repeat exploratory laparotomy was performed to investigate the possibility of sepsis resulting from a distal stump leak or bowel gangrene. However, no abdominal source of sepsis was found, and the serum lactate levels remained elevated. To further investigate the patient's condition, a contrast-enhanced CT (CECT) of the abdomen and thorax, as well as a non-contrast CT (NCCT) of the brain, were conducted. The results showed multiple liver and brain metastases (Figures 2, 3).

Because of the brain metastases, the patient became dependent on a ventilator, and the raised serum lactate levels were a result of extensive metastases secondary to the Warburg effect. Unfortunately, the patient passed away on the eighth day after the surgery.

## Discussion

Hyperlactatemia and lactic acidosis occur when there is either an excess production of lactate or a decreased utilization of it. Type A and type B are two main types of lactic acidosis. Type A lactic acidosis is caused by tissue hypoperfusion or severe hypoxemia, whereas type B lactic acidosis is associated with mechanisms such as accelerated glycolysis, drugs and toxins, or enzymatic deficiencies. Various diseases, including diabetes mellitus, thiamine deficiency, sepsis, liver disorders, and lactogenic cancer, are associated with type B lactic acidosis [2].

The initial association between malignancy and Type B lactic acidosis was reported in cases of acute leukemia [4]. Subsequently, it has been more frequently observed in hematological malignancies. Although rare, it has also been documented in solid tumors such as small-cell lung cancer, cholangiocarcinoma, breast cancer, gynecologic cancers, and metastases from unknown primary carcinomas [4].

The underlying cause of hyperlactatemia in solid malignancy is not yet fully understood. The newer theory suggests an upregulation of enzymes and transporters that facilitates glycolysis and lactate production. The accelerated glycolysis and lactate generation in patients with malignancy is known as the “Warburg effect.” This effect is observed in well-oxygenated patients and has been known to us since 1923 [5]. Its exact significance in tumor etiopathogenesis and progression is not fully understood. One of the other accepted hypotheses of hyperlactatemia in solid malignancy is hepatic dysfunction secondary to liver metastasis. Malignancy-associated lactic acidosis is commonly observed in patients with live metastasis supporting the hypothesis. However, the markers of liver function tests are mostly within normal limits in these individuals, and in some cases liver metastasis may not be present, casting doubt on this hypothesis.

Lactate generated through glycolysis does not lead to acidosis unless the Krebs or Cori cycles are affected. Therefore, hyperlactatemia may exist on its own, and the term lactic acidosis should be used when the pH level is below 7.35 and the plasma lactate concentration is over 5-6 mmol/L [6]. In cases of hyperglycemia and increased muscular catabolism, only lactate production rises, and no accompanying lactic acidosis is detected. Nevertheless, lactogenic tumors hinder pyruvate dehydrogenase (PDH), disrupting the Krebs and Cori cycles and resulting in lactic acidosis.

Aggressive chemotherapy is effective in correcting hyperlactatemia and associated acidosis. It is the only treatment that works in an otherwise helpless situation. The levels of lactate begin declining within 15 hours, but they may take up to three days to work [3]. Intravenous bicarbonates are also given in this condition, but the aim is to control the acidosis. The acidosis is known to induce respiratory fatigue and hemodynamic instability, which can be controlled with intravenous bicarbonates [7]. However, it does not affect the tumors and can further complicate the clinical situation with hypernatremia and hyperosmolality. Decreasing lactate by intravenous insulin has also been postulated but should be used in experimental conditions only [8].

The association between solid tumors and the Warburg effect is infrequent. In a recent review of lactic acidosis in solid malignancy, only 57 cases have been reported to date, which is a small number to draw any conclusions regarding the effect of surgery in these cases [9]. The case we are describing here portrays a pessimistic picture of surgery's role in such cases. However, this outcome should not discourage surgical intervention as it is the only way to eliminate other causes of hyperlactatemia, particularly in emergencies. In cases where lactate levels fail to normalize, further investigation is necessary as there may be other reasons for hyperlactatemia or lactic acidosis in the postoperative period. Overall, lactogenic tumors are highly aggressive and linked with a high mortality rate.

## Conclusions

Colonic malignancies rarely cause hyperlactatemia, even in the presence of hepatic metastasis. The removal of the primary tumor may not lead to a decrease in lactate levels. Elevated lactate levels may complicate postoperative management, which can potentially increase because of sepsis or raised abdominal compartment pressure. Knowledge of this condition can streamline further management, as palliative therapy may be sufficient.
